# Supplementary material for: Identification of photoperiod-induced specific miRNAs in the adrenal glands of Sunite sheep (Ovis aries)
Source: Front Vet Sci. 2022 Jul 22;9:888207. doi: 10.3389/fvets.2022.888207 (PMC9354845; doi:10.3389/fvets.2022.888207)
Supplement: Supplementary file 1 [file Data_Sheet_1.ZIP › Supplementary file/miRDeep2 file.zip!/miRDeep2 file/Know_Structure/SP42A1/oar-mir-107.pdf]

miRBase precursor : oar-mir-107
Total read count : 5857
oar-miR-107 read count : 5851
remaining reads : 6

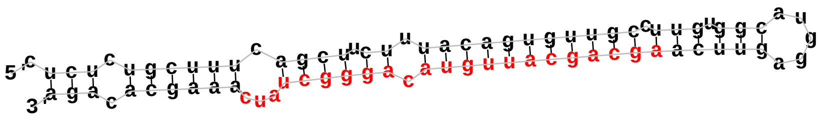

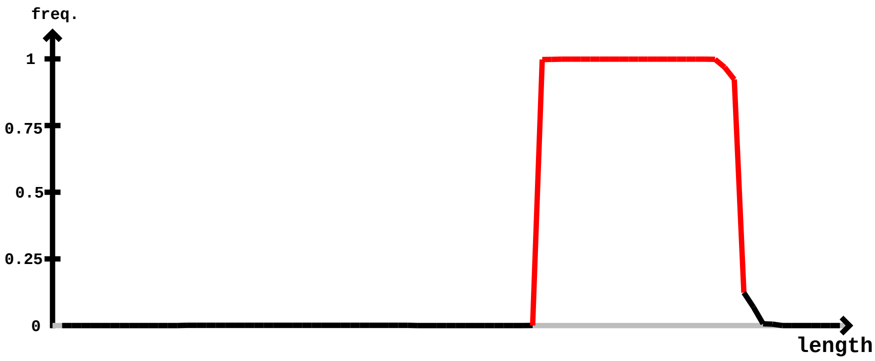

oar-miR-107

5'- cucucugcuuucagcuucuuuacaguguugccuuguggcauggaguucaagcagcauuguacagggcuaucaaagcacaga -3'	exp		
.(((.((((((.((((.((.(((((((((((.(((.(((.....))))))))))))))))).))))))...)))))).)))	reads	mm	sample
............agcuucuuuacagugCugccuug..	1	1	seq
............agcuucuuuacagugCugccuugu...	5	1	seq
....aagcagcauuguacagggcuau...........	3	0	seq
....agcagcauuguacagggc..............	4	0	seq
....agcagcauuguacagggcu.............	174	0	seq
....agcagcauuguacagggcua............	267	0	seq
....agcagcauuguacagggcuau...........	4675	0	seq
....agcagcauuguacagggcuauc..........	320	0	seq
....agcagcauuguacagggcuauca.........	364	0	seq
....agcagcauuguacagggcuaucaa........	8	0	seq
....agcagcauuguacagggcuaucaaa.......	2	0	seq
....agcagcauuguacagggcuauGaaa.......	26	1	seq
....gcagcauuguacagggcuau...........	3	0	seq
....cagcauuguacagggcu.............	1	0	seq
....cagcauuguacagggcuau...........	1	0	seq
....cagcauuguacagggcuauGaaa.......	3	1	seq
